# Comparison of Self-Conducted and Assistant-Supervised Uroflowmetry Methods

**DOI:** 10.7759/cureus.22030

**Published:** 2022-02-08

**Authors:** Serkan Dogan

**Affiliations:** 1 Urology, Sancaktepe Training and Research Hospital, Istanbul, TUR

**Keywords:** pvr, qave, qmax, assistant-supervised, self-conducted, uroflowmetry

## Abstract

Objective

This study aims to compare the results and patient satisfaction scores between uroflowmetry performed under the patient's control and assistant-supervised conventional uroflowmetry.

Methods

A total of 120 patients who had previous experience with uroflowmetry were included in the study. Patients were evaluated in two even groups of 60 patients each - those not receiving medical treatment (group 1) and those receiving medical treatment (group 2). Maximum flow rate (Qmax), average flow rate (Qave), voided volume, voiding time, post-void residual volume (PVR), and patient satisfaction survey scores were compared between the two separate uroflowmetry methods.

Results

There was a significant difference between assistant-supervised and self-conducted uroflowmetry in terms of Qmax, Qave, voiding time, and patient satisfaction scores. While comparing all patients, no significant difference was found in terms of PVR and voiding volume values. There was a significant difference in Qave, Qmax, PVR, and voiding time in both assistant-supervised and self-conducted uroflowmetry. As for voided volume, there was no significant difference between the groups in either procedure. When groups were evaluated within themselves, in group 1, there was a significant difference in voided volume, Qave, and PVR, while there was no significant difference in Qmax and voiding time. In Group 2, there was a significant difference in voided volume, Qave, and PVR, although there was no significant difference in Qmax and voiding time.

Conclusion

Maximum urine flow rate and mean urine flow rate measured by self-conducted uroflowmetry are higher than assistant-supervised (conventional) uroflowmetry, which can ensure patient privacy.

## Introduction

Uroflowmetry is a commonly used noninvasive urodynamic test that evaluates urine flow rate and pattern. It is increasingly performed in urology clinics [1]. The most critical parameters of this test are the maximum flow rate (Qmax) and flow pattern. A minimum volume of 150 mL is required for meaningful evaluation. Repeated testing is recommended when results show abnormal parameters or volume is insufficient [2].

Diagnostic accuracy of uroflowmetry is relatively variable to determine bladder outlet obstruction and is majorly affected by threshold values. Qmax below 10 mL/s has 70% specificity and 47% sensitivity, while >15 mL/s does not completely rule out bladder outlet obstruction [3]. Low Qmax may be associated with bladder outlet obstructions, decreased detrusor voiding contraction, and low bladder capacity [4,5]. The diagnostic value of the test is limited as it cannot differentiate underlying mechanisms. Specificity can be developed with repeated flow rate tests. Uroflowmetry can be used to follow up treatment results and evaluate objective findings [6]. For an ideal uroflowmetry measurement, rooms that are clean, provide privacy, are not very noisy, are close to the waiting area, and, if possible, connected to the examination room should be preferred. It is very important for patients to feel comfortable because the pelvic floor muscles need to be completely relaxed in order to properly empty the bladder.

In recent years, assistance-free uroflowmetry devices of various brands and models have been introduced. In the conventional uroflowmetry procedure, an assistant gives the patient commands to have the test done. The patient is then asked to empty the container. In uroflowmetry, the flow can be measured through the gravimetric method that is known as weighted measurement or rotary disc method. In our study, both uroflowmetry tests were performed with the gravimetric method. Complete privacy and self-control are preferred, especially in patients with previous knowledge and experience of uroflowmetry. Therefore, in this study, we compared uroflowmetry results between patients who completed assistant-supervised uroflowmetry and those who had the uroflowmetry test in complete privacy and under their own control. 

## Materials and methods

As a result of the power analysis for the study, 120 male patients were included in the study. Patients were selected from patients over 60 years of age with complaints of prostatism. Patients with diseases affecting neurologic function such as diabetes mellitus, receiving diuretic treatment, a history of urinary surgery, ongoing urinary infection, and a history of the urethral catheter were excluded from the study. Patients underwent all the necessary urological tests. Renal function tests, urine analyses, and prostate-specific antigen levels were recorded. While 60 patients were not receiving medical treatment, the other 60 were selected among those using alpha-blockers or a combination of alpha-blocker + 5α reductase inhibitor. When study power was taken as 85, the standard deviation of 10, and the minimum significant difference rate of 25 for independent groups in the Minitab® package software, it was found that 30 participants were required for each group. Two groups were formed for this procedure, which was used as a diagnostic and follow-up test. Group 1 consisted of patients not receiving medical treatment, while group 2 consisted of patients receiving medical treatment.

**Figure 1:**
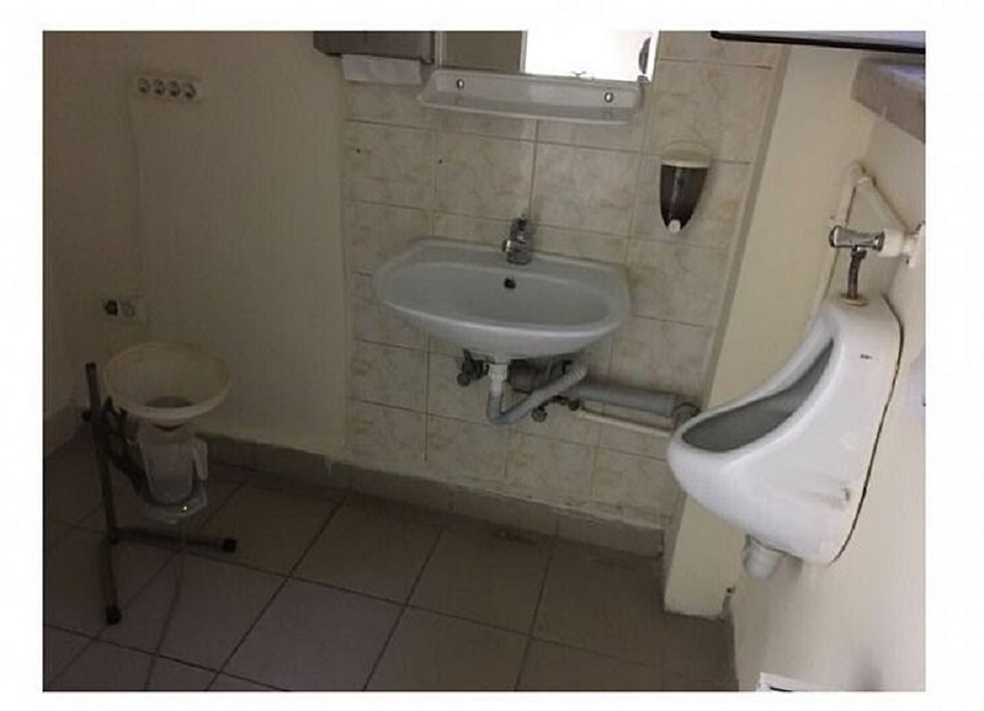
MMS, Flowstar uroflowmetry MMS - medical measurement systems

Both devices were calibrated before the study to provide standardization.* *Patients underwent uroflowmetry twice, with assistant-supervised uroflowmetry first and then self-conducted uroflowmetry, or self-conducted first and assistant-supervised uroflowmetry later, in random order. All procedures were performed in the standing position. Voided volume (ml), Qmax (ml/s), voiding time (s), and post-void residual volume (PVR) results of both tests were recorded and statistically analyzed and compared. For standard assistant-supervised uroflowmetry, medical measurement systems (MMS) brand Flowstar (FLWSTR-01; Mississauga, Ontario, Canada) model cabled and gravimetric measuring device was used (Figure 1).

**Figure 2:**
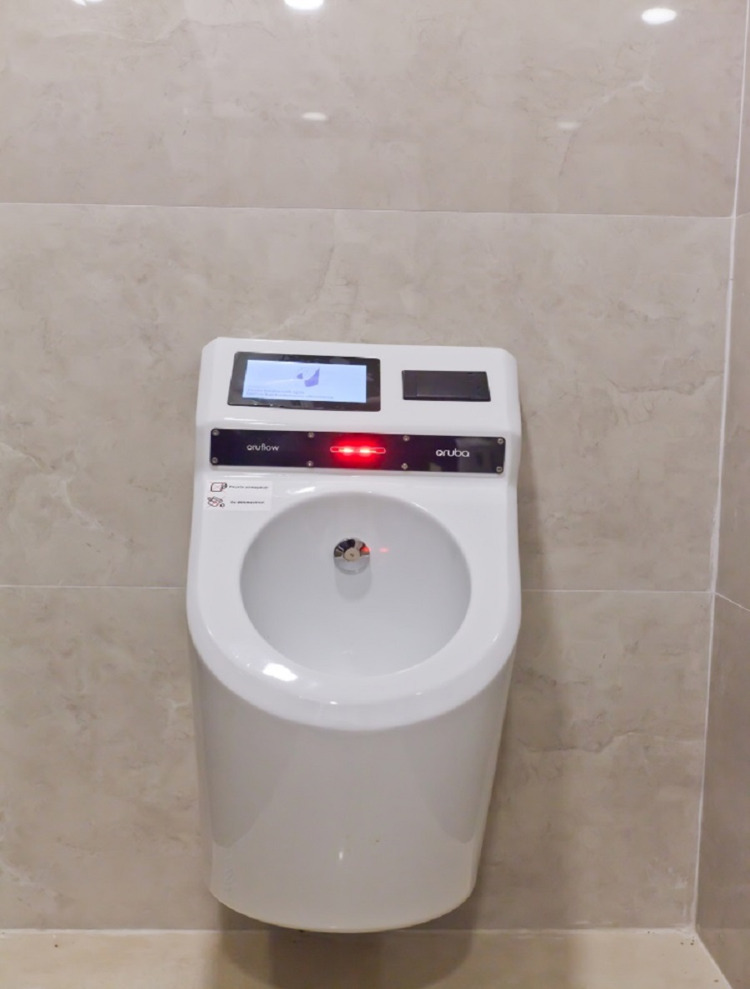
Oruflow uroflowmetry, Oruba Medical Technology

The process was performed in a locked room separated into two parts with a separator. There was a chair for the assistant and a unit including the processor of the device and a printer in the entrance. On the other side of the separator, there was the uroflowmetry device, a wall urinal to empty the container, and a sink for handwashing. The start and end of the procedure command were given from the other side of the separator. The results were printed on A4 paper at the end of the procedure. In the self-conducted process, the Oruba Medical Technology (Ankara, Turkey) brand Oruflow model, a wireless device that performs gravimetric measurements and wirelessly transmits results, was used (Figure 2).

For this procedure, patients entered a locked room and pressed a button to start the process when they felt ready, and the device would automatically end the process when there was no flow for five seconds and transmit results. The device also provides results printed on thermal paper with an integrated printer. As the equipment is shaped like a urinal, patients did not need to empty the urine container or control it beforehand. PVR was measured after the procedure for all patients. PVR measurement was performed with the Mindray North America (Mahwah, USA) brand Mindray DC-8 EXP ultrasonographic device, and three-dimensional volume measurement was made using an abdomen probe.

Patients were asked to fill out a form to evaluate their satisfaction from two aspects. This form was not the classical short patient satisfaction scale, but it was rather a form modified by us based on feedback about the use of the device. The patients were asked to answer six questions and to score them between 0-5 points. Satisfaction increased as the scores increased. The patient's level of satisfaction with privacy was also asked (Table 1).

**Table 1:** Patient satisfaction form

	Strongly disagree	Disagree	Uncertain	Slightly agree	Agree	Completely agree
Health care providers are good about explaining the reason of medical tests	0	1	2	3	4	5
I am sufficiently informed about the uroflowmeter test to be performed	0	1	2	3	4	5
My healthcare provider was friendly and I was able to ask questions freely	0	1	2	3	4	5
Using the uroflowmetry was easy	0	1	2	3	4	5
The uroflowmetry area was clean	0	1	2	3	4	5
I was comfortable in terms of privacy	0	1	2	3	4	5

Patients were informed of the study, and written consent was obtained. The study was approved by the Ethics Committee in September 2018, decision number 3312. The study was conducted in line with the ethical principles of the Declaration of Helsinki.

Statistical analysis was conducted using SPSS (Statistical Package for Social Sciences for Windows version 22.0, IBM Inc., Armonk, USA). The normality of the data was analyzed with the Kolmogorov-Smirnov test. According to the descriptive assessment of data with the Shapiro-Wilk test for both groups separately and in total, all parameters showed normal distribution except for PVR values. Paired Student's t-test was used to analyze data that included numerical results obtained from dependent subjects in the total assessment. Wilcoxon signed test was used in the comparison of PVR, which showed non-normal distribution. Student's t-test for independent samples was used for comparison of independent groups since there was numerical data with a normal distribution. Mann-Whitney U test was used for PVR comparison. P-values <0.05 were considered statistically significant.

## Results

The study included 120 patients. Patients were divided into two groups of 60 patients - those not receiving (group 1) and receiving medical treatment (group 2). The mean patient age of the groups was 62.93±5.21 years and 62.81±4.74 years, respectively, and there was no statistically significant difference (p=0.89) between them. When patients were evaluated in total, there was a statistically significant difference in voiding time, Qmax, average flow rate (Qave), and satisfaction scale score between the assistant-supervised and self-conducted uroflowmetry results (Table 2).

**Table 2:** Comparison of the measured values of all patients in two types of uroflowmetry Qmax - maximum flow rate; Qave - average flow rate; PVR - post-void residual Student's paired test; Wilcoxon signed test

	Assistant-supervised uroflowmetry	Self-conducted uroflowmetry	p-value
Voided volume (mL)	226.4±38.7	193.1±56.6	0.276
Qave (mL/s)	6.6±1.3	6.9±1.4	<0.001
Qmax (mL/s)	9.7±2.2	10.1±2.1	<0.001
PVR (cc)	67.0±28.9	66.4±26.3	0.318
Voiding time (sec)	34.5±9.1	35.9±9.4	0.012
Patient satisfaction score	19.1±3.4	22.4±2.7	<0.001

No significant difference was found in terms of PVR and voided volume values. Especially the questions about privacy, cleanliness, and ease of use were evaluated for both methods. Although no statistical comparison was made, scores given by the patients in the self-conducted group were higher in all three questions.

When the two groups were compared, there was a significant difference in Qave, Qmax, PVR, and voiding time in both assistant-supervised and self-conducted uroflowmetry results. There was no significant difference in voided volume between the groups (Table 3). 

**Table 3:** Comparison of the measured values of groups in two types of uroflowmetry Qmax - maximum flow rate; Qave - average flow rate; PVR - post-void residual Student's paired test; Wilcoxon signed test

		Group 1	Group 2	p-value
	Age	62.8±4.7	62.9±5.2	0.89
Self-conducted uroflowmetry	Voided volume (ml)	224.1±54.7	220.3±58.9	0.744
	Qave (ml/s)	7.5±1.3	6.2±1.2	<0.001
	Qmax (ml/s)	10.2±1.4	9.4±1.3	<0.001
	PVR (cc)	48.5±14.0	62.3±30.1	<0.001
	Voiding time (sec)	31.6±5.3	40.1±10.7	<0.001
Assistant-supervised uroflowmetry	Voided volume (ml)	200.6±46.4	198.2±52.5	0.735
	Qave (ml/s)	7.1±1.3	5.9±1.2	<0.001
	Qmax (ml/s)	10.2±1.3	9.1±1.4	<0.001
	PVR (cc)	55.5±15.2	78.6±34.4	<0.001
	Voiding time (sec)	30.8±4.7	38.1±10.9	<0.001

When the groups were compared in terms of the parameters measured by both methods, there was a significant difference in group 1 in voided volume, Qave, and PVR, while no significant difference was found in Qmax and voiding time. Similarly, there was a significant difference in Qave and PVR, while no significant difference was found in Qmax and voiding time in group 2 (Table 4).

**Table 4:** Comparison of two types of uroflowmetry results in two groups Qmax - maximum flow rate; Qave - average flow rate; PVR - post-void residual Student's paired test; Wilcoxon signed test

	Group 1			Group 2		
	Assistant-supervised uroflowmetry	Self-conducted uroflowmetry	p-value	Assistant-supervised uroflowmetry	Self-conducted uroflowmetry	p -value
Voided volume (mL)	200.6±46.4	224.1±54.7	p≤0.001	198.2±52.5	220.3±58.9	p≤0.001
Qave (mL/s)	7.1±1.3	7.5±1.3	p≤0.001	5.9±1.2	6.2±1.2	p≤0.001
Qmax (mL/s)	10.2±1.3	10.2±1.4	0.813	9.1±1.4	9.4±1.3	0.186
PVR (cc)	55.5±15.2	48.5±14.0	p≤0.001	78.6±34.4	62.3±30.1	p≤0.001
Voiding time (s)	30.8±4.7	31.6±5.3	0.057	38.1±10.9	40.1±10.7	0.102

## Discussion

Uroflowmetry is the most commonly used noninvasive test in the diagnosis and follow-up of lower urinary system disorders [7]. Uroflowmetry results are not the symptom- and dysfunction-specific. A significant decrease in flow rate in uroflowmetry is observed in many pathologies such as bladder outlet obstruction, neurogenic bladder, urethral stenosis, and detrusor under-activity. There was no study in the literature comparing two separate methods using different devices for this important investigation. In the current study, patient satisfaction scores, Qmax, and Qave values were significantly higher in the self-conducted method. Patients may experience worse uroflowmetry results than urinating at home for various reasons such as excessive urgency, embarrassment, and anxiety. With such studies, it is aimed to obtain the most optimal results by reducing some of these causes. These results will be beneficial both for the correct diagnosis of the patients and for giving the appropriate treatment to the clinicians.

Uroflowmetry results are affected by patient comfort, sufficient urine volume, and even circadian rhythm; therefore, multiple tests are recommended instead of using a single test result with the minimum required urine volume of 150 ml. The most important uroflowmetry parameter is Qmax [8,9]. In addition to uroflowmetry, PVR is also part of this assessment. According to the European Association of Urology Guidelines, PVR should be used in routine practice and as a decision-making tool for surgical treatment [10]. Excess pressure on the patients also affects uroflowmetry results. Okcelik et al. compared PVR measurement following uroflowmetry with PVR measured at any given time and found that PVR measurement following uroflowmetry was significantly higher [11].

We designed this study because patient comfort may affect results. Obtained data showed that patient comfort positively affects both uroflowmetry and PVR results. We think that uroflowmetry, which patients perform alone and without command, simulates normal life better. The fact that the device they are voiding is a regular urinal, which is also more successful in reflecting normal situations. We obtained patient survey results that support our hypothesis. As an advantage, we see that the patient starts the test when he feels ready. There were many patients unable to start urination after the "start" command in the assisted test. In some patients, we encountered the problem of starting before the command.

It is also known that procedures requiring personnel also increase expenses. Studies have been previously conducted on these subjects. Mombelli et al. conducted a study initially with 121 patients and ended with 100 patients, in which patients first underwent conventional uroflowmetry at the hospital and later performed simple uroflowmetry at home; when results were compared, they found that at-home procedure had higher Qave, but was not statistically significant. In conclusion, they indicated that there were similar reliability and efficacy at a lower cost [12]. Porru et al. compared home-measured and institute-measured uroflowmetry results and found no statistically significant difference and even suggested that repeated measurement performed at home with circadian rhythm was both natural and beneficial [13]. In this study, both Qave and Qmax were statistically significantly higher when all patients were evaluated. Terai et al. presented a device they developed on their own, though there was no statistical comparison using patient data. This study was remarkable, as it emphasized the importance of increased personnel cost and patient privacy. They also mentioned the limitations of measuring flow rate [14]. In our study, an assistant was not needed for self-conducted uroflowmetry. However, a cost analysis was not carried out. Patient privacy was provided as specified in the survey. The patients in the self-conducted uroflowmetry group had better scores in terms of privacy. In addition, the cleanliness of the uroflowmetry room obtained better results in the self-conducted group. Twenty-seven patients who gave ≤3 points to the cleanliness in the assisted group were questioned. The patients generally attributed these low points to dissatisfaction with emptying the urine container to the toilet and malodor. Six patients who gave low points in the self-conducted uroflowmetry group stated the reasons related to the sink and malodor. 

This study found it to be an advantage that an assistant is not required for the device. Patients had no problem after brief information about using the device. As a result of the study, statistically better data were obtained from self-conducted uroflowmetry.

Study limitations include the fact that we did not investigate the anxiety levels of the patients. Although it is subjective data, this was a limitation of our study. We should also mention that this study expands horizons by being the first of its kind as well as indicates the need for further research on this subject. Finally, we could not perform sub-group statistical analysis since the number of questions was small.

## Conclusions

Our study is the first to compare two different uroflowmetry methods, both of which were performed in a hospital environment. In conclusion, we observed that patient privacy, the comfort of being alone, and self-control of the test created a statistically significant difference in test results. The assistant supervised method requires operator assistance in initiating the test, emptying the urine container, concluding the test. We think that this situation causes loss of workforce.

## References

[1] Homma Y, Gotoh M, Kawauchi A (2017). Clinical guidelines for male lower urinary tract symptoms and benign prostatic hyperplasia. Int J Urol.

[2] Kranse R, van Mastrigt R (2003). Causes for variability in repeated pressure-flow measurements. Urology.

[3] Reynard JM, Yang Q, Donovan JL (1998). The ICS-'BPH' Study: uroflowmetry, lower urinary tract symptoms and bladder outlet obstruction. Br J Urol.

[4] Idzenga T, Pel JJ, van Mastrigt R (2008). Accuracy of maximum flow rate for diagnosing bladder outlet obstruction can be estimated from the ICS nomogram. Neurourol Urodyn.

[5] Siroky MB, Olsson CA, Krane RJ (1979). The flow rate nomogram: I. development. J Urol.

[6] Siroky MB, Olsson CA, Krane RJ (1980). The flow rate nomogram: II. clinical correlation. J Urol.

[7] Ochiai A, Kojima M (1998). Correlation of ultrasound-estimated bladder weight with ultrasound appearance of the prostate and postvoid residual urine in men with lower urinary tract symptoms. Urology.

[8] Keskin MZ, Karaca E, Uçar M, Ateş E, Yücel C, İlbey YÖ (2020). Comparison of uroflowmetry tests performed with a sensation of normal desire to void versus urgency and correlation of test results with IPSS. Turk J Urol.

[9] Shoukry I, Susset JG, Elhilali MM, Dutartre D (1975). Role of uroflowmetry in the assessment of lower urinary tract obstruction in adult males. Br J Urol.

[10] Gravas S, Cornu JN, Gacci M (2020). Management of mon-neurogenic male LUTS. European Association of Urology.

[11] Okçelik S, Soydan H, Ateş F, Yilmaz Ö, Malkoç E, Şenkul T, Karademir K (2018). Correlation between residual volume of male patients after uroflowmetry and random residual volume. Low Urin Tract Symptoms.

[12] Mombelli G, Picozzi S, Messina G, Truffelli D, Marenghi C, Maffi G, Carmignani L (2014). Free uroflowmetry versus "Do-It-Yourself" uroflowmetry in the assessment of patients with lower urinary tract symptoms. Int Urol Nephrol.

[13] Porru D, Scarpa RM, Prezioso D, Bertaccini A, Rizzi CA (2005). Home and office uroflowmetry for evaluation of LUTS from benign prostatic enlargement. Prostate Cancer Prostatic Dis.

[14] Terai A, Ueda N, Utsunomiya N, Kohei N, Aoyama T, Inoue K (2006). Automatic switching and guidance system to facilitate unassisted uroflowmetry using commercial electronic devices. Int J Urol.

